# Chromoblastomycosis in Peru: a retrospective review of 13 cases

**DOI:** 10.1186/s12879-025-11475-4

**Published:** 2025-08-28

**Authors:** Mercedes Sanchez-Diaz, Nicolas Antunez de Mayolo, Cesar Ramos, Omayra Chincha, Beatriz Bustamante

**Affiliations:** 1 Facultad de Medicina “Alberto Hurtado”, Universidad Peruana Cayetano Heredia, Lima, 15102 Peru; 2 Instituto de Medicina Tropical Alexander von Humboldt, Universidad Peruana Cayetano Heredia, Lima, 15102 Peru; 3 Departamento de Enfermedades Infecciosas, Tropicales y Dermatológicas, Hospital Nacional Cayetano Heredia, Lima, Peru

**Keywords:** Chromoblastomycosis, *Fonsecaea sp*., Subcutaneous mycosis, Neglected-tropical disease, Tropical dermatology

## Abstract

**Background:**

Chromoblastomycosis (CBM) is a chronic subcutaneous mycosis caused by dematiaceous fungi that mainly affects rural workers in tropical and subtropical regions. The disease poses a treatment challenge due to its refractory nature and high relapse rate. To date, few cases have been reported in Peru.

**Methods:**

We retrospectively reviewed epidemiological, clinical, microbiological, and treatment data from CBM cases diagnosed between 2011 and 2024 at the Clinical Mycology Unit of the Instituto de Medicina Tropical Alexander von Humboldt (IMTAvH) in Lima, Peru. Diagnosis was confirmed by the identification of muriform cells either on direct microscopic examination or histopathology.

**Results:**

Of the 15 identified cases, 13 had sufficient data for inclusion. In summary, 84% of the patients were men, 77% acquired the disease in the Peruvian Amazon jungle (predominantly in Ucayali and San Martin), the median age was 65.3 years (range: 33–85), and the average disease duration was 10.7 years (range: 1–25 years). The lower extremities were most frequently affected (53%), followed by the upper extremities (38%). Plaque-like and verrucous lesions were the most common (38% each), whereas tumoral and cicatricial forms were less frequent (15% each). The aetiological agent was identified by morphology in nine patients. *Fonsecaea sp.* was the most frequently identified pathogen (46%), followed by *Cladophialophora sp.* (15%) and *Phialophora sp.* (7%). Nine patients (69%) received oral itraconazole (200–400 mg/day) combined with cryosurgery; two (15%) received itraconazole alone; and two (15%) received itraconazole combined with terbinafine (500 mg/day). Treatment duration ranged from 5 to 136 months, with six patients (46%) achieving a cure.

**Conclusions:**

CBM in Peru is likely underdiagnosed and underestimated due to low disease recognition, limited diagnostics in remote areas, and lack of mandatory reporting.

## Background

Chromoblastomycosis (CBM) is a chronic subcutaneous mycosis caused by dematiaceous (melanized) fungi and is classified as a Neglected Tropical Disease (NTD) [1]. The disease occurs in tropical and subtropical regions, including Latin America, Africa, and Asia [2]. In Latin America, most cases are reported in Brazil, Mexico, and Venezuela [3–8].

CBM primarily affects men aged 30–50 years who are exposed to contaminated soil and plant material. Lesions are polymorphic in appearance and may present as nodular, tumoral, verrucous, plaque-like, or cicatricial forms, typically involving the feet and legs [9, 10]. The most prevalent pathogens belong to the genus *Fonsecaea spp.* [9, 11]. Diagnosis is based on muriform cell identification on microscopic examination or histopathology. However, diagnosis is often delayed due to limited access to healthcare in endemic areas, particularly in developing countries. Treatment is challenging because CBM shows a poor response to antifungal therapy, and interventions like surgical excision and adjuvant physical therapies (e.g., cryotherapy) are commonly required [12, 13].

In Peru, fewer than 10 cases have been reported in the past 30 years [14–19]. Given the country’s extensive tropical and subtropical jungle regions, this small number likely reflects underreporting, as CBM is not a notifiable disease. This study aimed to describe CBM cases treated at a referral hospital in Lima, Peru, including their epidemiological and clinical characteristics, treatment data, and outcomes.

## Methods

We conducted a retrospective review of CBM cases diagnosed at the Clinical Mycology Unit of the Instituto de Medicina Tropical Alexander von Humboldt (IMTAvH) from January 2011 to December 2024. All patients received treatment and regular follow-up care at Hospital Nacional Cayetano Heredia, a tertiary referral centre in Lima, Peru. The study protocol was approved by the Institutional Review Board of Universidad Peruana Cayetano Heredia. This study was conducted in accordance with the Declaration of Helsinki.

Diagnosis was based on the identification of muriform cells, either by direct microscopic examination of skin scrapings with 10% potassium hydroxide (KOH) or by histopathology. Fungal cultures were performed in all cases using Sabouraud Dextrose Chloramphenicol Agar, incubated at 27 °C, and monitored for up to 4 weeks. PCR was not performed routinely but was conducted in two cases. We collected data on demographics, clinical characteristics, microbiological and histopathological findings, treatment, and outcomes from clinical records from the Mycology Unit and Hospital Nacional Cayetano Heredia.

Lesions were classified according to Carrion’s proposed clinical types: nodular, tumoral, cicatricial, plaque, or verrucous forms [20]. Disease severity was graded according to the number, extent and dissemination of lesions, following the criteria proposed by Queiroz-Telles [13]: mild (single plaque or nodule < 5 cm in diameter), moderate (one or more lesions covering < 15 cm), and severe (extensive cutaneous involvement) [13].

## Results

### Epidemiological and clinical characteristics

**Fig. 1:**
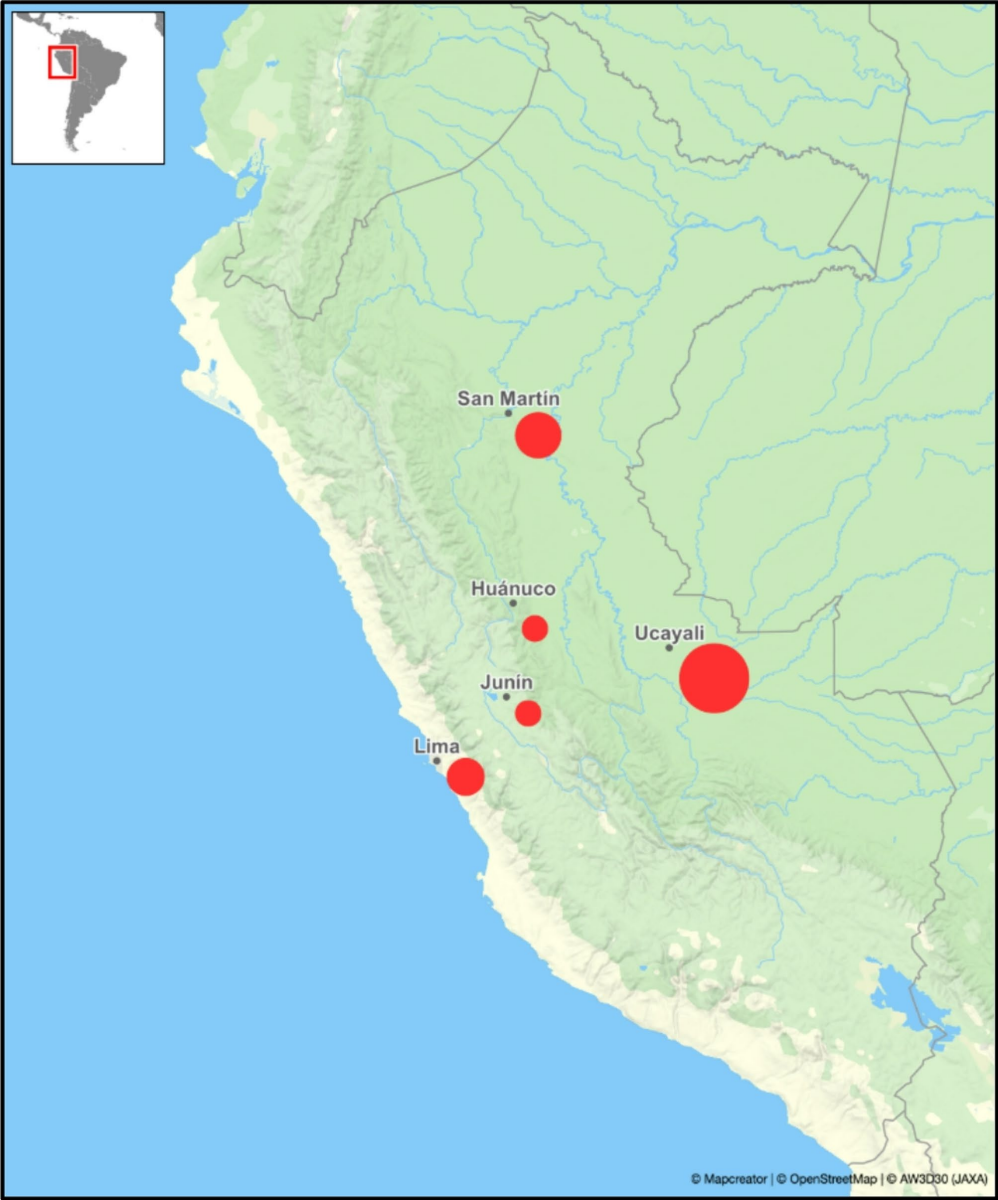
Geographical distribution of patients with CBM in Peru

Of the 15 cases, 13 had sufficient clinical data for analysis. Most patients were men (84%), with a male-to-female ratio of 5.5:1. The median age was 65.3 years (± 11.2; range 33–85 years). Most patients (77%) acquired the disease in the Peruvian Amazon jungle, mainly in Ucayali (46%) and San Martin (23%) (Fig. 1). All patients reported occupational exposure to soil, although only seven (53%) recalled a history of trauma. Comorbidities included diabetes mellitus (2 cases) and arterial hypertension (1 case).

**Fig. 2:**
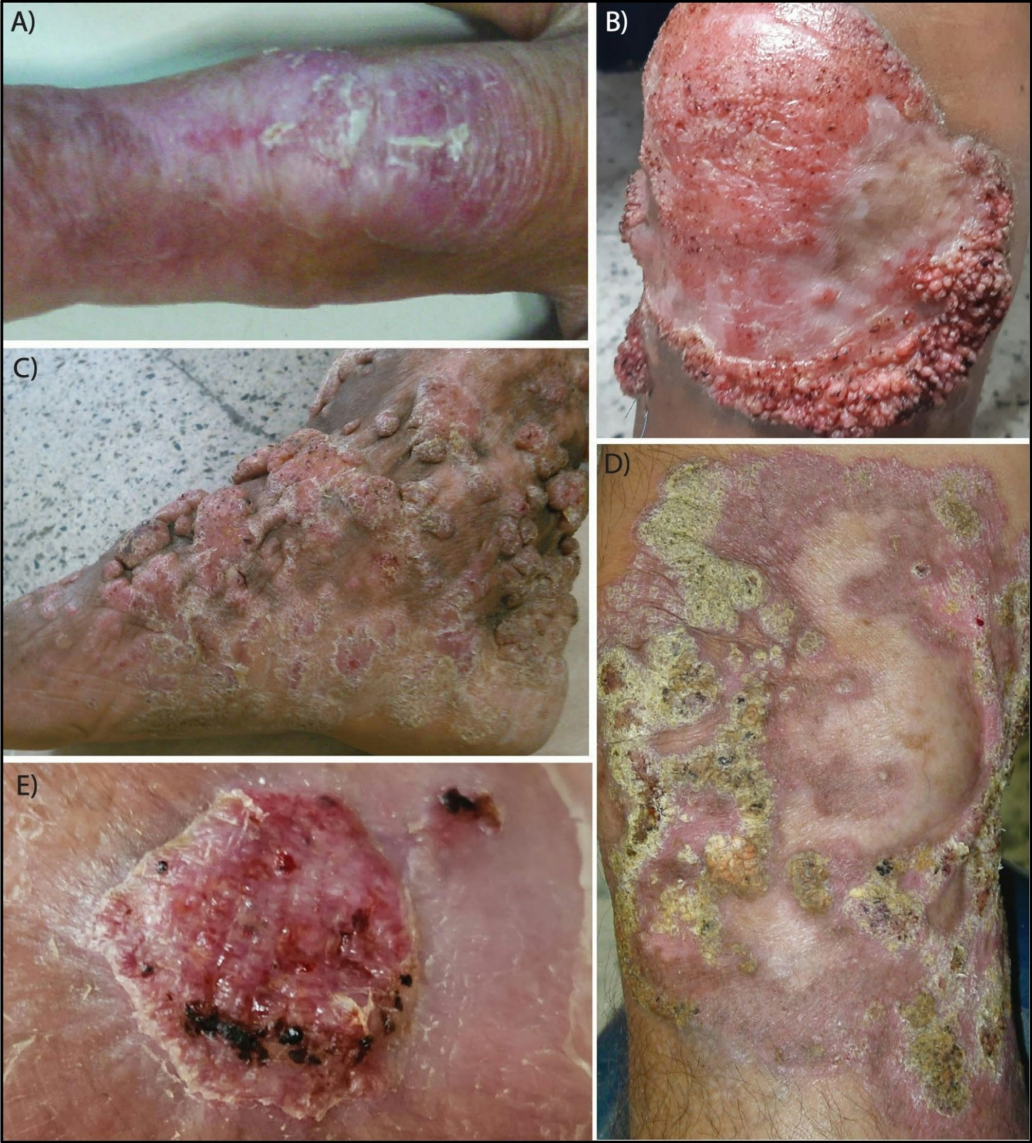
Clinical presentation of chromoblastomycosis. **A** Plaque-type lesion on the middle finger. **B** Verrucous lesion on the knee. **C** Tumoral lesion on the right foot. **D** Cicatricial lesion with an active verrucous peripheral border and central scarring. **E** Nodular lesion on the dorsum of the foot

The average disease duration was 10.7 years (range: 1–25). The lower extremities were most frequently affected (53%), followed by the upper extremities (38%). Lesion size ranged from 2 to 50 cm, and six patients (46.1%) had a single lesion. Four of seven patients with lower extremity involvement reported inconsistent use of protective footwear during occupational activities. Disease severity was mild in five patients (38%), moderate in three (23%), and severe in five (38%). Plaque-like and verrucous lesions were the most common presentations (38% each), followed by tumoral and cicatricial forms (15% each) (Fig. 2).

Pruritus was reported in ten patients (84%), pain in four (30%), and functional limitations affecting work-related activities in four (30%). Eight patients (61%) had received antifungal treatments, including itraconazole, fluconazole, ketoconazole, amphotericin B, or topical agents, with minimal or no improvement before referral. One patient (Case 1) had been previously misdiagnosed with leishmaniasis and tuberculosis. Four patients reported using topical herbal remedies without any clinical benefit.

### Microbiological diagnosis

Muriform cells were observed by direct microscopy in 12 of 13 patients (92%). Pigmented fungi were observed in all cultures. The pathogen was identified at genus level by morphology in nine cases: *Fonsecaea sp.* (six cases), *Cladophialophora sp.* (two cases), and *Phialophora sp.* (one case). Additionally, PCR was performed in two cases, both of which confirmed *F. pedrosoi*. Histopathological examinations were performed in 11 cases; all showed muriform cells. Additional findings included pseudoepitheliomatous hyperplasia (9/11), diffuse granulomatous inflammation with epithelioid histiocytes, lymphocytes, plasma cells, and multinucleated giant cells around muriform cells (9/11), suppurative foci (neutrophilic abscesses) (6/11), and dermal fibrosis (4/11).

### Treatment and follow-up

Outcomes were documented for 11 patients. Nine (69%) patients received oral itraconazole (200–400 mg/day) in combination with cryosurgery. Two (15%) received itraconazole 200 mg/day alone; and two patients (15%) received a combination of itraconazole 200 mg/day, terbinafine 500 mg/day and cryosurgery. Treatment duration ranged from 5 to 136 months.

Six (46%) patients achieved clinical cure. All mild or moderate cases were cured within one year, with no recurrence during short-term follow-up (< 1 year). Two patients died of unrelated causes during treatment but showed improvement. Two patients (Cases 3 and 7) with severe, extensive disease remain on long-term therapy at the moment (78 and 136 months). Patient characteristics and treatment outcomes are summarised in Table 1.

**Table 1:** Clinical-Epidemiological profile and treatment outcomes of patients with CBM

Clinical-Epidemiological Profile and Treatment Outcomes of CBM cases												
Patient	Age/Sex	Region of origin	Occupation	Trauma history	Duration of disease (years)	Isolated species†	Clinical Form	Site of infection	Grade of severity	Treatment regimen (mg/day)	Treatment duration (months)	Follow-up
1	65/F	Ucayali	Farmer	No	6	ND	Verrucous	Forearm	Mild	TZ 200 + CRY	3	Improved*
2	89/M	Junin	Farmer	No	10	ND	Verrucous, cicatricial	Leg and tight	Severe	ITZ 200 + CRY	6	Relapse^§^
3	49/M	Ucayali	Farmer	Yes	9	F. pedrosoi†	Verrucous	Leg	Severe	ITZ 200 + CRY	78^‡^	Improved
4	68/M	Ucayali	Farmer	Yes	25	Cladophialophora sp.	Tumoral	Leg and foot	Severe	ITZ 200 + TRB 500	24	Failure, lost follow-up
5	80/M	Huanuco	Farmer	No	16	Fonsecaea sp.	Verrucous	Leg	Severe	ITZ 200	-	Lost follow-up
6	66/M	Lima	Carpenter	Yes	3	Fonsecaea sp.	Plaque	Arm	Mild	ITZ 200 + CRY	12	Cure
7	41/M	Ucayali	Farmer	Yes	21	Fonsecaea sp.	Verrucous, cicatricial	Leg	Severe	ITZ 400 + TRB 500 + CRY + TMT	136^‡^	Improved
8	62/M	Ucayali	Farmer	No	20	Fonsecaea sp.	Plaque	Forearm	Moderate	ITZ 200 + CRY	11	Cure
9	61/M	Ucayali	Gardener	No	1	ND	Plaque	Trunk	Mild	ITZ 200 + CRY	5	Cure
10	33/M	San Martin	Farmer	No	4	Phialophora sp.	Plaque	Leg	Moderate	ITZ 200 + CRY	11	Cure
11	68/M	San Martin	Farmer	Yes	8	F. pedrosoi†	Plaque	Third finger	Mild	ITZ 200 + CRY	8	Cure
12	85/M	Lima	Carpenter	Yes	10	ND	Tumoral	Forearm	Moderate	ITZ 200 + CRY	6	Cure
13	83/F	San Martin	Farmer	Yes	6	Cladophialophora sp.	Nodular	Foot	Mild	ITZ 200	2	Improved*

Muriform cells were observed by direct microscopy in 12 of 13 patients (all except Case 9). Histopathological analysis was performed in 11 patients (all except Cases 4 and 13), confirming the presence of muriform cells in all examined samples

*ITZ* itraconazole, *CRY* cryosurgery, *TRB* terbinafine, *TMT* thermotherapy, *ND* not determined

*Died of unrelated causes during treatment

†Species identification to the genus level was based on morphological characteristics; PCR was additionally performed for species confirmation in Cases 3 and 11. ‡Received intermittent treatment. §Relapsed after 1 year and received a new course of oral antifungal therapy at another institution, final outcome not documented

## Discussion

This study reports chromoblastomycosis (CBM) casesdiagnosed between 2011 and 2024 at a tertiary referral centre in Lima, Peru. Two cases had been published previously; the others were unreported until this present study [17, 18].

Our demographic data are consistent with the literature: most cases occurred in adult men aged 40–70 with occupational or recreational exposure to soil, through which the fungus enters via traumatic implantation, with a predominance of lesions on the lower extremities [2–9]. All cases were chronic, with a mean disease duration of 10.7 years; the longest disease course lasted 25 years, and the shortest was 1 year. Delays in definitive diagnosis are linked to limited access to diagnostic tools (including laboratory equipment and trained personnel in rural areas), probable lack of disease awareness in endemic areas, and delayed healthcare seeking due to the slow progression of the disease, as outlined in the WHO Road Map for Neglected Tropical Diseases [21, 22]. The predominant symptom in our series was pruritus (84%), which could coexist with mild pain or tenderness. Pruritus is notable, as scratching may promote the spread of the fungi to adjacent body areas and facilitate secondary infection [23].

*Fonsecaea sp.* was the most common agent, as reported elsewhere in Latin America, where *F. pedrosoi* accounts for 90% of isolates [5]. Interestingly, *Cladophialophora sp.*, typically found in arid and semi-arid climates [9], was isolated in two cases from humid rainforest regions (Ucayali and San Martin). The occurrence of this pathogen in the Peruvian jungle has been previously reported [14].

In early stages, small, well-delimited lesions can be excised surgically, with good success rates and minimal recurrence, either alone or in combination with antifungal agents [24–26]. However, as disease severity increases, CBM lesions become refractory to treatment. Treatment of CBM is challenging because data on drug effectiveness and regional susceptibility patterns are limited. Therefore, therapy selection often relies on data from open clinical studies and expert opinion.

CBM management typically requires prolonged antifungal therapy, can be costly, and is often difficult to access in resource-limited settings [22]. Systemic therapeutic options include itraconazole, terbinafine, voriconazole, and posaconazole [27]. Itraconazole is considered the most effective treatment option for inoperable lesions, with response rates ranging from 15 to 80%, depending on disease severity and the causative agent [5, 9, 12, 13, 26]. However, in vitro resistance has been reported in patients on long-term treatment [28]. Terbinafine is the second most commonly used antifungal, particularly in Asia [29, 30]. Severe, long-standing cases often present with lymphedema, fibrosis, and reduced vascularisation, leading to lower drug bioavailability. Refractory cases benefit from dual therapy with itraconazole and terbinafine due to their synergistic effect [31].

Adjuvant therapies, such as cryotherapy, thermotherapy (local heat), topical imiquimod, photodynamic therapy, CO2 laser treatment, and 5-fluorouracil, can also be beneficial [32–35]. At our centre, treatment usually combines oral itraconazole with cryotherapy sessions administered at our hospital. The use of cryosurgery is not standardized and is based on the treating physician’s assessment, considering lesion type, extent, and treatment response. Cryotherapy with liquid nitrogen has proven to be cost-effective and to shorten the duration of systemic azole therapy [36–38].

Our study is limited by its retrospective design and small number of cases, as it reflects the experience of a single referral centre. CBM in Peru is likely underdiagnosed, and its true prevalence is probably underestimated due to the lack of disease recognition among healthcare workers, limited diagnostic capacity in remote areas of Peru, and the absence of mandatory reporting and epidemiological surveillance.

## Conclusions

Chromoblastomycosis (CBM) occurs sporadically in the Peruvian jungle, particularly in Ucayali, but its true burden is likely to be underestimated. *Fonsecaea sp.* was the most common pathogen. Lack of awareness and frequent misdiagnosis probably contribute to delayed diagnosis. Most patients benefited from combined treatment with systemic antifungal agents, such as itraconazole, and physical therapies, such as cryosurgery. Further research is needed to improve treatment strategies.

## Data Availability

The datasets generated or analyzed during this study are available from the corresponding author upon request.

## Abbreviations

CBM: Chromoblastomycosis
ITZ: Itraconazole
CRY: Cryosurgery
TRB: Terbinafine
TMT: Thermotherapy
ND: Not determined

## References

[1] Neglected. Accessed on May 7 tropical diseases 2025 [https://www.who.int/health-topics/neglected-tropical-diseases#tab=tab_1]

[2] Santos DWCL, de Azevedo C, de MPES, Vicente VA, Queiroz-Telles F, Rodrigues AM, de Hoog GS, et al. The global burden of chromoblastomycosis. PLoS Negl Trop Dis. 2021;15(8):e0009611. 34383752 10.1371/journal.pntd.0009611 PMC8360387

[3] Barroeta S, Mejía de Alejos A, Franco de Arias C, Prado A, Zamora R. Cromomicosis En El Estado de Lara. Dermatol Venez. 1988;24(2):134–37.

[4] Silva JP, de Souza W, Rozental S. Chromoblastomycosis: a retrospective study of 325 cases on Amazonic region (Brazil). Mycopathologia. 1998;143(3):171–5. 10.1023/a:1006957415346 10353215

[5] Bonifaz A, Carrasco-Gerard E, Saúl A. Chromoblastomycosis: clinical and mycologic experience of 51 cases. Mycoses. 2001;44(1–2):1–7. 10.1046/j.1439-0507.2001.00613.x 11398635

[6] Navarrete MR, Arenas R, Estrada VFM, Diéguez CEA, Mayorga J, Bonifaz A, et al. Chromoblastomycosis in mexico. Review of 603 cases, during seven decades. Dermatol Cosmet Med Quir. 2014;12(2):87–93.

[7] Santos DWCL, Vicente VA, Weiss VA, de Hoog GS, Gomes RR, Batista EMM, et al. Chromoblastomycosis in an endemic area of Brazil: a clinical-epidemiological analysis and a worldwide haplotype network. J Fungi. 2020;6(4): 204. 10.3390/jof6040204 PMC7711792 33022951

[8] Barbosa LSP, Souza YRC, Sasaki CS, Santos DWD, Rossato L. Chromoblastomycosis in brazil: A review of 450 published cases. Rev Soc Bras Med Trop. 2024;57:e00205–2024. 39570152 10.1590/0037-8682-0132-2024 PMC11654747

[9] Queiroz-Telles F, de Hoog S, Santos DWCL, Salgado CG, Vicente VA, Bonifaz A, et al. Chromoblastomycosis. Clin Microbiol Rev. 2017;30(1):233–76. 10.1128/CMR.00032-16 PMC5217794 27856522

[10] Queiroz-Telles F, Nucci M, Colombo AL, Tobón A, Restrepo A. Mycoses of implantation in Latin America: an overview of epidemiology, clinical manifestations, diagnosis and treatment. Med Mycol. 2011;49(3):225–36. 10.3109/13693786.2010.539631 21128710

[11] Torres-Guerrero E, Isa-Isa R, Isa M, Arenas R. Chromoblastomycosis. Clin Dermatol. 2012;30(4):403–8. 10.1016/j.clindermatol.2011.09.011 22682188

[12] Restrepo A, Gonzalez A, Gomez I, Arango M, de Bedout C. Treatment of chromoblastomycosis with itraconazole. Ann N Y Acad Sci. 1988;544:504–16. 10.1111/j.1749-6632.1988.tb40448.x 2850755

[13] Queiroz-Telles F, Purim KS, Fillus JN, Bordignon GF, Lameira RP, Van Cutsem J, et al. Itraconazole in the treatment of chromoblastomycosis due to Fonsecaea Pedrosoi. Int J Dermatol. 1992;31(11):805–12. 1330949 10.1111/j.1365-4362.1992.tb04252.x

[14] Cavero J, Delgado V. Cromoblastomicosis Por cladosporium Sp. Folia Dermatol Peru. 2004;15:28–31.

[15] Solórzano S, García R, Hernández-Córdova G. Cromomicosis: reporte de Un Caso incapacitante. Rev Peru Med Exp Salud Publica. 2011;28(3):552–5. 22086640 10.1590/s1726-46342011000300023

[16] Ventura-Flores R, Failoc-Rojas V, Silva-Díaz H. Chromoblastomycosis: clinical and microbiological characteristics of a neglected disease. Rev Chilena Infectol. 2017;34(4):404–7. 10.4067/s0716-10182017000400404 29165523

[17] Carcamo PM, Schwalb A, Seas C. Chromoblastomycosis. A case of a verrucous plaque from the tropics. Am J Trop Med Hyg. 2020;103(2):547–8. 10.4269/ajtmh.20-0243 PMC7410413 32758344

[18] Schwalb A, Seas C. Chromoblastomycosis. N Engl J Med. 2020;383(2):e7. 10.1056/NEJMicm1913199 32640136

[19] Galarza-Manyari C. Enfoque de Las micosis profundas En El Perú. Dermatol Peru. 1996;6(1 suppl):S39-40.

[20] Carrion AL. Chromoblastomycosis. Ann N Y Acad Sci. 1950;50(10):1255–82. 10.1111/j.1749-6632.1950.tb39826.x 14783318

[21] World Health Organization. Ending the neglect to attain the sustainable development goals: a road map for neglected tropical diseases 2021–2030. Genève, Switzerland: World Health Organization; 2021.

[22] Smith DJ, Queiroz-Telles F, Rabenja FR, Hay R, Bonifaz A, Grijsen ML, et al. A global chromoblastomycosis strategy and development of the global chromoblastomycosis working group. PLoS Negl Trop Dis. 2024;18(10):e0012562. 39405322 10.1371/journal.pntd.0012562 PMC11478817

[23] Esterre P, Queiroz-Telles F. Management of chromoblastomycosis: novel perspectives. Curr Opin Infect Dis. 2006;19(2):148–52. 16514339 10.1097/01.qco.0000216625.28692.67

[24] Valentin J, Grotta G, Muller T, Bourgeois P, Drak Alsibai K, Demar M, et al. Chromoblastomycosis in French Guiana: epidemiology and practices, 1955–2023. J Fungi. 2024. 10.3390/jof10030168 10.3390/jof10030168 PMC10971162 38535177

[25] Mouchalouat MF, Gutierrez Galhardo MC, Zancopé-Oliveira RM, Monteiro Fialho PC, de Oliveira Coelho JMC, Silva Tavares PM, et al. Chromoblastomycosis: a clinical and molecular study of 18 cases in Rio de janeiro, Brazil. Int J Dermatol. 2011;50(8):981–6. 21781072 10.1111/j.1365-4632.2010.04729.x

[26] Yang CS, Chen CB, Lee YY, Yang CH, Chang YC, Chung WH, et al. Chromoblastomycosis in Taiwan: a report of 30 cases and a review of the literature. Med Mycol. 2018;56(4):395–405. 10.1093/mmy/myx075 29087525

[27] Criado PR, Careta MF, Valente NYS, Martins JEC, Rivitti EA, Spina R, et al. Extensive long-standing chromomycosis due to Fonsecaea pedrosoi: three cases with relevant improvement under voriconazole therapy. J Dermatol Treat. 2011;22(3):167–74. 10.3109/09546630903585074 20666671

[28] Andrade TS, Castro LGM, Nunes RS, Gimenes VMF, Cury AE. Susceptibility of sequential *Fonsecaea pedrosoi* isolates from chromoblastomycosis patients to antifungal agents. Mycoses. 2004;47(5–6):216–21. 10.1111/j.1439-0507.2004.00984.x 15189187

[29] Esterre P, Inzan CK, Ramarcel ER, Andriantsimahavandy A, Ratsioharana M, Pecarrere JL, et al. Treatment of chromomycosis with terbinafine: preliminary results of an open pilot study. Br J Dermatol. 1996;134(Suppl 46):33–6. 8763467 10.1111/j.1365-2133.1996.tb15658.x

[30] Esterre P, Inzan CK, Rtasioharana M, Andriantsimahavandy A, Raharisolo C, Randrianiaina E, et al. A multicenter trial of terbinafine in patients with chromoblastomycosis: effect on clinical and biological criteria. J Dermatol Treat. 1998;9(Suppl 1):S29–34.

[31] Gupta AK, Taborda PR, Sanzovo AD. Alternate week and combination itraconazole and terbinafine therapy for chromoblastomycosis caused by *Fonsecaea pedrosoi* in Brazil. Med Mycol. 2002;40(5):529–34. 10.1080/mmy.40.5.529.534 12462534

[32] Pérez-Blanco M, Hernández Valles R, García-Humbría L, Yegres F. Chromoblastomycosis in children and adolescents in the endemic area of the Falcón state, Venezuela. Med Mycol. 2006;44(5):467–71. 16882614 10.1080/13693780500543238

[33] Lyon JP, Pedroso e Silva Azevedo CM, Moreira LM, de Lima CJ, de Resende MA. Photodynamic antifungal therapy against chromoblastomycosis. Mycopathologia. 2011;172(4):293–7. 21643843 10.1007/s11046-011-9434-6

[34] de Sousa Mda, Belda GT, Spina W Jr, Lota R, Valente PR, Brown NS. Topical application of imiquimod as a treatment for chromoblastomycosis. Clin Infect Dis. 2014;58(12):1734–7. 24633683 10.1093/cid/ciu168 PMC4036686

[35] Hu Y, Qi X, Sun H, Lu Y, Hu Y, Chen X, et al. Photodynamic therapy combined with antifungal drugs against chromoblastomycosis and the effect of ALA-PDT on *Fonsecaea* in vitro. PLoS Negl Trop Dis. 2019;13(10): e0007849. 10.1371/journal.pntd.0007849 PMC6850556 31671098

[36] Castro LGM, Pimentel ERA, Lacaz CS. Treatment of chromomycosis by cryosurgery with liquid nitrogen: 15 years’ experience. Int J Dermatol. 2003;42(5):408–12. 10.1046/j.1365-4362.2003.01532.x 12755986

[37] Kullavanijaya P, Rojanavanich V. Successful treatment of chromoblastomycosis due to *Fonsecaea pedrosoi* by the combination of itraconazole and cryotherapy. Int J Dermatol. 1995;34(11):804–7. 10.1111/j.1365-4362.1995.tb04404.x 8543418

[38] Bonifaz A, Martínez-Soto E, Carrasco-Gerard E, Peniche J. Treatment of chromoblastomycosis with itraconazole, cryosurgery, and a combination of both. Int J Dermatol. 1997;36(7):542–7. 10.1046/j.1365-4362.1997.00085.x 9268758

